# Downregulation of epithelial DUOX1 in chronic obstructive pulmonary disease

**DOI:** 10.1172/jci.insight.142189

**Published:** 2021-01-25

**Authors:** Caspar Schiffers, Cheryl van de Wetering, Robert A. Bauer, Aida Habibovic, Milena Hristova, Christopher M. Dustin, Sara Lambrichts, Pamela M. Vacek, Emiel F.M. Wouters, Niki L. Reynaert, Albert van der Vliet

**Affiliations:** 1 Department of Pathology and Laboratory Medicine, Larner College of Medicine, University of Vermont, Burlington, Vermont, USA.; 2 Department of Respiratory Medicine, NUTRIM School of Nutrition and Translational Research in Metabolism, Maastricht University Medical Center, Maastricht, Netherlands.; 3 Department of Medical Biostatistics, Larner College of Medicine, University of Vermont, Burlington, Vermont, USA.; 4 Ludwig Boltzman Institute for Lung Health, Vienna, Austria.

**Keywords:** Cell Biology, Pulmonology, COPD, Extracellular matrix

## Abstract

Chronic obstructive pulmonary disease (COPD) is a chronic respiratory disease characterized by small airway remodeling and alveolar emphysema due to environmental stresses such as cigarette smoking (CS). Oxidative stress is commonly implicated in COPD pathology, but recent findings suggest that one oxidant-producing NADPH oxidase homolog, dual oxidase 1 (DUOX1), is downregulated in the airways of patients with COPD. We evaluated lung tissue sections from patients with COPD for small airway epithelial DUOX1 protein expression, in association with measures of lung function and small airway and alveolar remodeling. We also addressed the impact of DUOX1 for lung tissue remodeling in mouse models of COPD. Small airway DUOX1 levels were decreased in advanced COPD and correlated with loss of lung function and markers of emphysema and remodeling. Similarly, DUOX1 downregulation in correlation with extracellular matrix remodeling was observed in a genetic model of COPD, transgenic SPC-TNF-α mice. Finally, development of subepithelial airway fibrosis in mice due to exposure to the CS-component acrolein, or alveolar emphysema induced by administration of elastase, were in both cases exacerbated in *Duox1*-deficient mice. Collectively, our studies highlight that downregulation of DUOX1 may be a contributing feature of COPD pathogenesis, likely related to impaired DUOX1-mediated innate injury responses involved in epithelial homeostasis.

## Introduction

Chronic obstructive pulmonary disease (COPD) is a chronic irreversible disease of the lungs characterized by airflow limitation due to destruction of the lung parenchyma (emphysema) and/or remodeling of the small airways (1, 2). COPD is a major and growing global health problem that is predicted to be the third leading cause of death worldwide by 2030 (3). In susceptible individuals, environmental insults such as cigarette smoke (CS) are at the foundation of COPD pathogenesis, which is characterized by persistent inflammation and a protease/antiprotease imbalance, collectively contributing to alveolar destruction and airway remodeling (4). Importantly, small airway disease and emphysema development may mechanistically be linked, since CS-induced small airway inflammation may propagate to the alveolar septa, in turn destroying bronchiolar-alveolar attachments, and eventually proceed into lung parenchymal destruction (5).

Although classically thought to be independent pathological manifestations of COPD, more recent evidence indicates that more emphysematous lungs tend to have fewer small airways (6). In fact, the disappearance of small airways, which begins in the early stages of COPD, is a dominant characteristic in all patients with COPD and appears to precede emphysema development (7).

A well-documented aspect of COPD is the presence of an oxidant/antioxidant imbalance (8), believed to be caused by ROS present in tobacco smoke or produced during chronic inflammation. This imbalance is illustrated by increased irreversible oxidation of critical biological molecules (9), evidence of mitochondrial dysfunction, and aging-related impairment in antioxidant defense mechanisms (10), and is thought to contribute to injury to critical cell constituents, lung cell dysfunction, and lung function decline (11, 12). Based on this premise, antioxidant treatment therapies have been advocated for COPD, with limited success. Although small molecule thiol antioxidants (e.g., N-acetyl cysteine, erdosteine) have shown some clinical benefit, this likely relates to their mucolytic properties rather than their proposed antioxidant effects (12, 13). Supplementation with other small molecular antioxidants has not shown any benefit and may even have adverse effects (14).

Contrasting the general concept of oxidative stress, regulated biological production of ROS by, e.g., NADPH oxidases (NOX) is increasingly implicated in diverse biological processes via so-called redox-based signaling (15). The NOX family of NADPH oxidases produce ROS (O_2_^-^ or H_2_O_2_) as their primary function to mediate critical physiological functions including host defense, cell proliferation, or differentiation (16). Although all 7 NOX enzymes are expressed in various cell types within the lung, relatively little is known with respect to their potential role in COPD pathology. Several reports indicate that NOX4 is upregulated in airway smooth muscle of patients with COPD and correlates with disease severity (17, 18). NOX2, primarily expressed in cells of the innate immune system, also appears to be increased in COPD, and some studies suggest that NOX2 contributes to experimental emphysema, although contrasting findings were reported as well and NOX2 deficiency may even promote spontaneous emphysema (19–21). Recently, a crucial role was reported for the NOX organizer protein NOXO1, which regulates the function of several isoforms, in CS-induced emphysema (21). In contrast to the general concept of increased involvement of NOX enzymes COPD pathology, recent studies indicate that the dual oxidases DUOX1 and, to a lesser extent, DUOX2 are downregulated within the bronchial epithelia of healthy smokers and patients with COPD (21–23). Both DUOX1 and DUOX2 are primarily expressed in airway and alveolar epithelia, with a proposed critical function in innate antimicrobial and antiviral host defense, with DUOX1 being particularly critical in innate airway epithelial wound responses to diverse nonmicrobial triggers (24, 25). In this regard, DUOX1 downregulation during COPD may conceivably contribute to disease progression or exacerbation, due to a decline in regenerative capacity and host defense. In addition, downregulation of epithelial DUOX1 was recently shown to promote features of epithelial-mesenchymal transition (EMT) (26), which may also be relevant for small airway remodeling in COPD (27).

The present study aimed to address the importance of DUOX1 downregulation in small airway remodeling as well as emphysema development in COPD. We observed that downregulation of airway DUOX1 in patients with COPD was strongly correlated with lung function loss, and with markers of small airway remodeling and destruction. Moreover, we provide evidence that DUOX1 deficiency leads to enhanced features of small airway remodeling and emphysema in experimental mouse models of COPD, suggesting that DUOX1 downregulation in COPD may actively contribute to disease pathogenesis.

## Results

### Airway epithelial DUOX1 was suppressed in patients with COPD and correlated with lung function.

**Figure 1:**
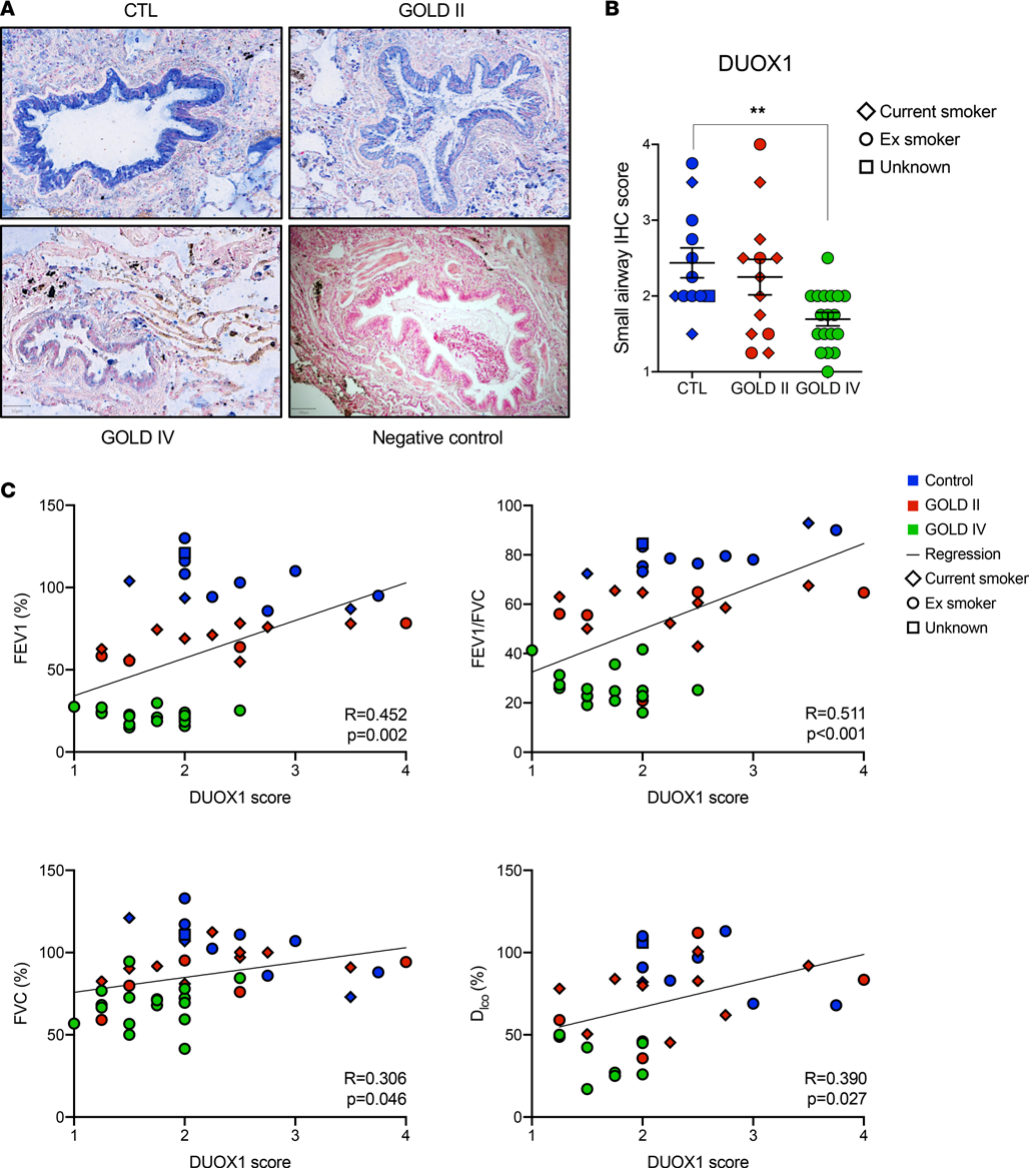
DUOX1 loss in small airways of patients with COPD is associated with loss of lung function, emphysema, and airway remodeling. (**A**) IHC for DUOX1 (blue) with Nuclear Fast Red counterstaining (red) in lung tissue of a representative control, GOLD II, and GOLD IV patient (including a negative control; original magnification, ×100). (**B**) Small airway score of DUOX1 in control, GOLD II, and GOLD IV patients. Data are shown as mean ± SEM. **P < 0.01, by 1-way ANOVA. (**C**) Pearson’s correlations (2-tailed significance) between DUOX1 scores and percentages of FEV1, FVC, and DLCO and FEV1/FVC ratio (determined in ref. 32) in all subjects studied. Colored labels highlight controls (blue), GOLD II (red), and GOLD IV (green) patients, and symbol shapes represent smoking status (current or former smokers). DUOX1, dual oxidase 1; COPD, chronic obstructive pulmonary disease; GOLD II, moderate COPD; GOLD IV, very severe COPD.

Following up on previous studies demonstrating that lung epithelial DUOX1 mRNA expression is attenuated in active smokers and in patients with COPD (22, 23), we evaluated tissue sections from a previous study cohort of patients with COPD and control subjects at Maastricht University (UM) (28) for protein expression of DUOX1 in the small airways. Control subjects and patients with COPD were age-matched, but the ratio of current to ex-smokers did significantly differ and patients with COPD had smoked more pack-years (Supplemental Table 1; supplemental material available online with this article; https://doi.org/10.1172/jci.insight.142189DS1). As expected, DUOX1 protein was prominently expressed in the bronchial and small airway epithelium, and small airway DUOX1 expression was found to be reduced in tissue sections from patients with COPD, especially in patients with very severe COPD (GOLD IV), compared with age-matched, non-COPD controls (Figure 1, A and B, with representative images of a scoring scale 1–4; Supplemental Figure 1). Adjustment for smoking status had no effect on the ANOVA results comparing control and GOLD stages (Supplemental Table 2). After correcting for age, sex, pack-years, and smoking status by ANCOVA analysis, small airway DUOX1 scoring revealed a clear trend towards significance (*P* = 0.058) between GOLD IV patients (mean ± SEM: 1.582 ± 0.210) and controls (mean ± SEM: 2.487 ± 0.262). We also examined the overall relationship between DUOX1 scores and pack-years of smoking and found no relationship (*r* = -0.083, *P* = 0.635) (Supplemental Table 3). The GOLD IV patients included in this study underwent lung volume reduction surgery (LVRS) because of severe emphysema (29), suggesting that DUOX1 downregulation may be associated with emphysema.

Correlation of epithelial DUOX1 staining scores with parameters of lung function indicated a striking positive association of DUOX1 score with spirometric parameters (FEV_1_, FVC) and diffusing capacity (Dlco, diffusing capacity for carbon monoxide, which indicates loss of alveolar surface area and capillary bed, impairing diffusion; ref. 30), both of which were based on inclusion of all subjects (including controls, Figure 1C and Table 1) and patients with COPD (GOLD II vs. GOLD IV) alone (Table 1). We examined the correlations between DUOX1 score and percentages of FEVI, DLCO, and FVC and FEV1/FVC ratio, after adjustment for covariates (smoking status, age, BMI), which had little effect on the results, although the correlation between DUOX1 score and DLCO was slightly attenuated (Supplemental Table 3). Because relationships of DUOX1 scores with percentages of FEVI, DLCO, FVC, and FEV1/FVC may not be linear, we also computed nonparametric Spearman’s correlation coefficients based on ranks rather than numerical values. The results were very similar to the Pearson’s correlations, indicating that the linearity assumption is reasonable. We also computed Pearson’s correlations using a logarithmic transformation of DUOX1 score. Again, the results were very similar to those based on the untransformed score (Supplemental Table 3).

Alterations in lung tissue content of elastin and collagen from these patients were previously published (28), showing that elastin was significantly decreased in patients with COPD in both alveolar and small airway walls, whereas collagen was found to be increased in both alveolar and small airway walls. DUOX1 staining scores measured in the present study positively correlated with the critical remodeling marker elastin in the small airways, although no statistically significant correlation was observed with collagen (Table 1). Overall, these results imply that the gradual loss of DUOX1 in the small airways of patients with COPD is associated with impaired lung function, emphysema, and airway remodeling in these patients.

### DUOX1 downregulation in mouse models of COPD was associated with increased remodeling.

**Figure 2:**
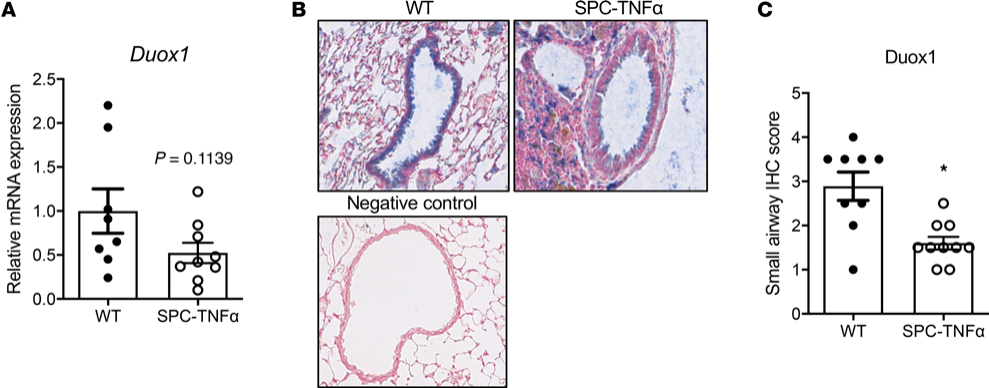
Airway epithelial DUOX1 is decreased in SPC-TNF-α mice. (**A**) Relative *Duox1* mRNA expression in lungs of 6-month-old SPC-TNF-α mice and littermate controls (*n* = 8–9 from 2 separate experiments; 2-tailed unpaired nonparametric *t* test). (**B** and **C**) Representative staining of DUOX1 protein (in blue; original magnification, ×400) with corresponding small airway IHC score of airway epithelial DUOX1 in SPC-TNF-α mice compared with WT littermate controls (*n* = 9–10). Data are shown as mean ± SEM. *P < 0.05, by 2-tailed unpaired *t* test. DUOX1, dual oxidase 1.

**Figure 3:**
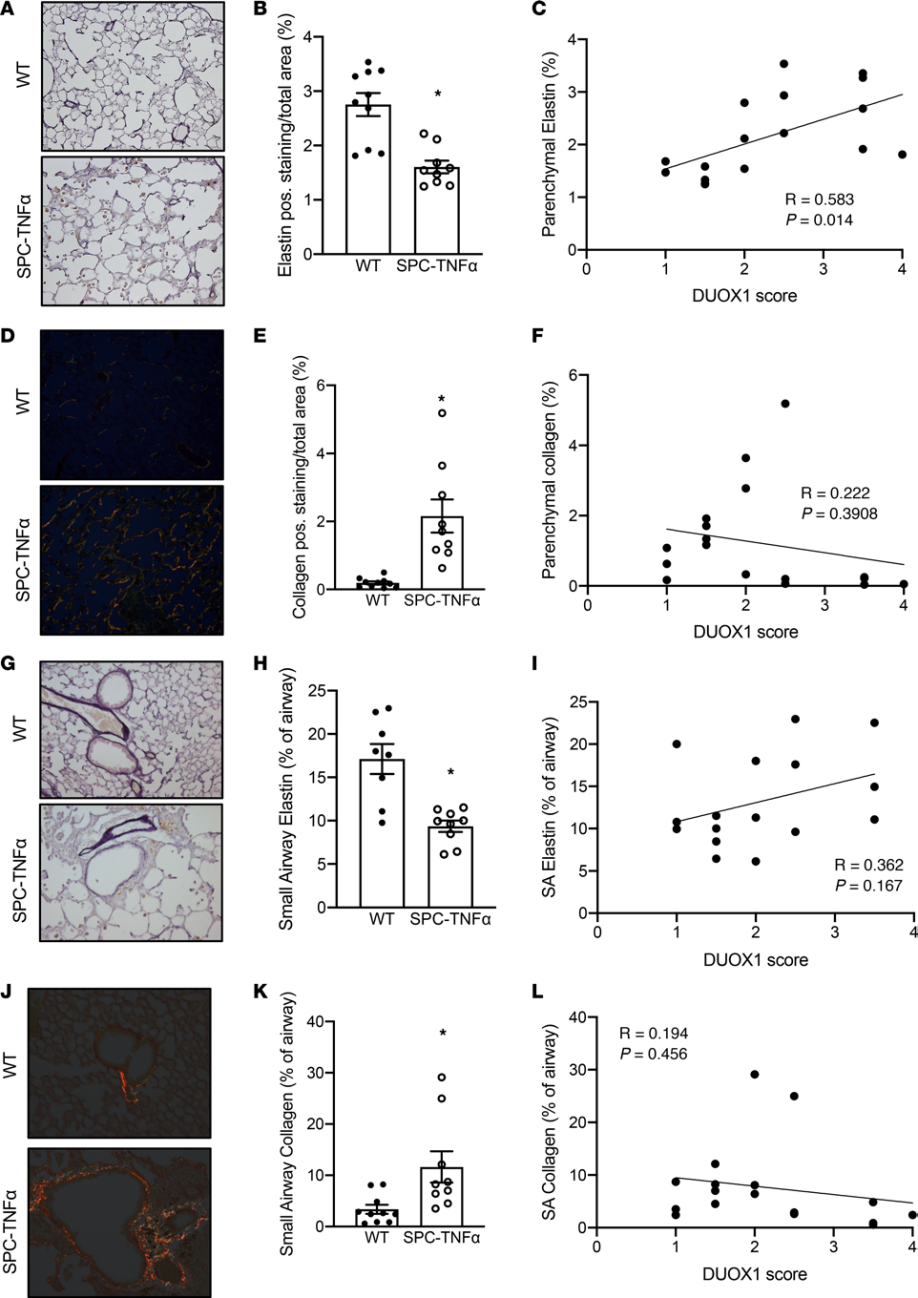
Airway epithelial DUOX1 loss in SPC-TNF-α mice is associated with elastin remodeling. (**A–L**) IHC analysis (original magnification, ×200) and quantification (percentage of surface area) of elastin and collagen in parenchyma (**A–F**) and small airways (**G–L**) in 6-month-old WT and SPC-TNF-α mice. Quantified stainings (*n* = 9–10) were correlated to small airway DUOX1 scores. Data are shown as mean ± SEM. *P < 0.05, by 2-tailed unpaired *t* test. DUOX1, dual oxidase 1.

We next investigated whether DUOX1 was similarly downregulated in mouse models of COPD. First, we evaluated lung tissues from SPC-TNF-α mice, transgenic mice that constitutively overexpress TNF-α under the surfactant protein C (SP-C) promoter. These mice develop chronic neutrophilic inflammation, airway remodeling (increased collagen deposition and elastin remodeling), and parenchymal alveolar destruction with increased expiratory static compliance, indicative of COPD development (28, 31–33). Consistent with our findings in patients with COPD, DUOX1 was downregulated in 6-month-old SPC-TNF-α mice compared with WT counterparts, indicated by a trend towards decreased *Duox1* mRNA expression (*P* = 0.09) (Figure 2A), and significantly decreased small airway DUOX1 protein levels (Figure 2, B and C). We assessed elastin and collagen remodeling in the parenchyma and small airways of these mice, which showed reduced levels of parenchymal and small airway elastin in SPC-TNF-α mice (Figure 3, A and B, and G and H) as well as increased collagen (Figure 3, D and E, and J and K). Furthermore, parenchymal elastin staining, but not small airway elastin levels, positively correlated with the DUOX1 score (Figure 3, C and I). No significant correlation was observed between parenchymal and small airway collagen levels and DUOX1 staining score (Figure 3, F and L). Collectively, these findings are consistent with observations in human COPD, showing an association between DUOX1 downregulation and elastin degradation.

We additionally utilized a mouse model of chronic exposure to acrolein, a major bioactive component of CS (34), which was previously shown to induce pulmonary injury and inflammation, mucus hypersecretion, and airflow limitation (35, 36). Consistent with observations of reduced *DUOX1* mRNA expression in the airways of smokers (22), chronic exposure of C57BL/6J mice to acrolein (5 ppm; 4 hours/day, for 2 weeks) resulted in reduced lung DUOX1 protein (Supplemental Figure 2A; see complete unedited blots in the supplemental material) and *Duox1* mRNA expression (Supplemental Figure 2B). Acrolein exposure also enhanced production of TGF-β1 (Supplemental Figure 2C), a profibrotic growth factor that is thought to contribute to features of EMT and subepithelial fibrosis in COPD (37–39). To address the potential impact of Duox1 downregulation for such EMT features, we isolated mouse tracheal epithelial cells (MTECs) from C57BL/6J mice and exposed them to either acrolein or TGF-β, over a 2-week period. Indeed, both acrolein and TGF-β1 caused a downregulation of *Duox1* mRNA levels (Supplemental Figure 2D), and acrolein exposure resulted in a dose-dependent loss of E-cadherin, with a concomitant gain in vimentin (Supplemental Figure 2E; see complete unedited blots in the supplemental material). Moreover, acrolein-induced alterations in E-cadherin and vimentin were enhanced in MTECs from *Duox^–/–^* mice, indicating that Duox1 deletion by acrolein may contribute to EMT. In agreement with the effects of acrolein, *DUOX1* mRNA was also downregulated upon 24-hour exposure of primary bronchial epithelial cells (PBECs) to freshly prepared cigarette smoke extract (CSE) (Supplemental Figure 2F).

**Figure 4:**
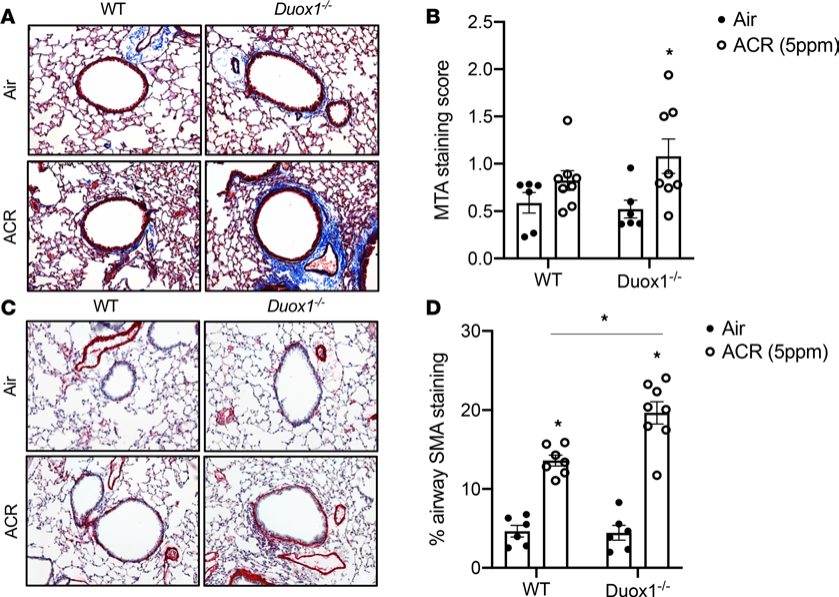
*Duox1* deficiency sensitizes airways to acrolein-induced peribronchiolar fibrosis. C57BL/6J mice were exposed to acrolein (ACR) and analyzed (original magnification, ×200) for collagen by Masson’s trichrome (**A** and **B**) or α-smooth muscle actin (**C** and **D**). Quantification of staining was based on 6–8 mice per group from 2 separate experiments. Data are shown as mean ± SEM. *P < 0.05, by 2-way ANOVA.

Chronic exposure of acrolein to mice also resulted in features indicative of enhanced peribronchiolar fibrosis (40), as illustrated by subepithelial collagen deposition (Figure 4, A and B) and enhanced α-smooth muscle actin staining (Figure 4, C and D). Importantly, these features of peribronchiolar fibrosis were enhanced in *Duox1^–/–^* mice, with acrolein-induced subepithelial collagen deposition being significant only in *Duox1^–/–^* mice and acrolein-induced peribronchial α-smooth muscle actin levels being significantly exacerbated in *Duox1^–/–^* mice. We examined lung tissue mRNA expression of several markers of inflammation and airway remodeling (Supplemental Figure 3). Acrolein exposure tended to increase *Il6, Il13,* and *Cxcl1* (murine KC) mRNA levels (albeit nonsignificantly) and significantly increased *Mmp9* expression. However, no significant differences were observed between WT and *Duox1^–/–^* mice. Collectively, these various findings suggest that lung DUOX1 expression is reduced in several mouse models of COPD, and our observations of acrolein-induced EMT and peribronchiolar fibrosis suggest that loss of DUOX1 may sensitize airways to these important hallmarks of COPD.

### Duox1 deficiency enhanced elastase-induced emphysema in mice.

**Figure 5:**
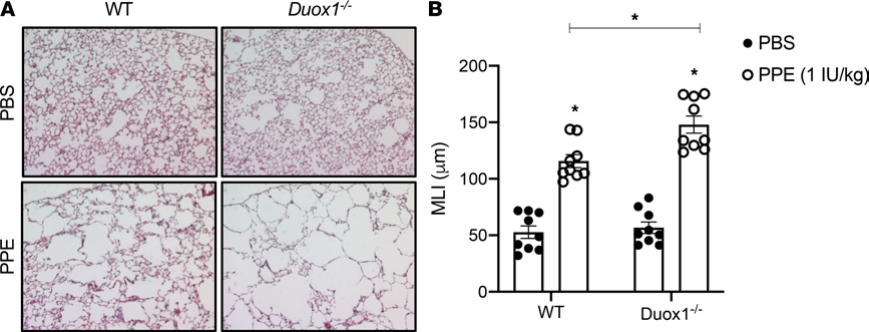
*Duox1* deficiency enhances development of elastase-induced emphysema. WT and *Duox1^–/–^* mice were exposed to 50 μL porcine pancreatic elastase (PPE) or PBS control, and lung tissues were analyzed by H&E staining (original magnification, ×100) for alveolar enlargement (**A**), with corresponding (**B**) calculation of alveolar mean linear intercept (MLI, μm) as a measure of emphysema. Data are shown as mean ± SEM; *n* = 9 per group, from 2 separate experiments. *P < 0.05 by 2-way ANOVA.

**Figure 6:**
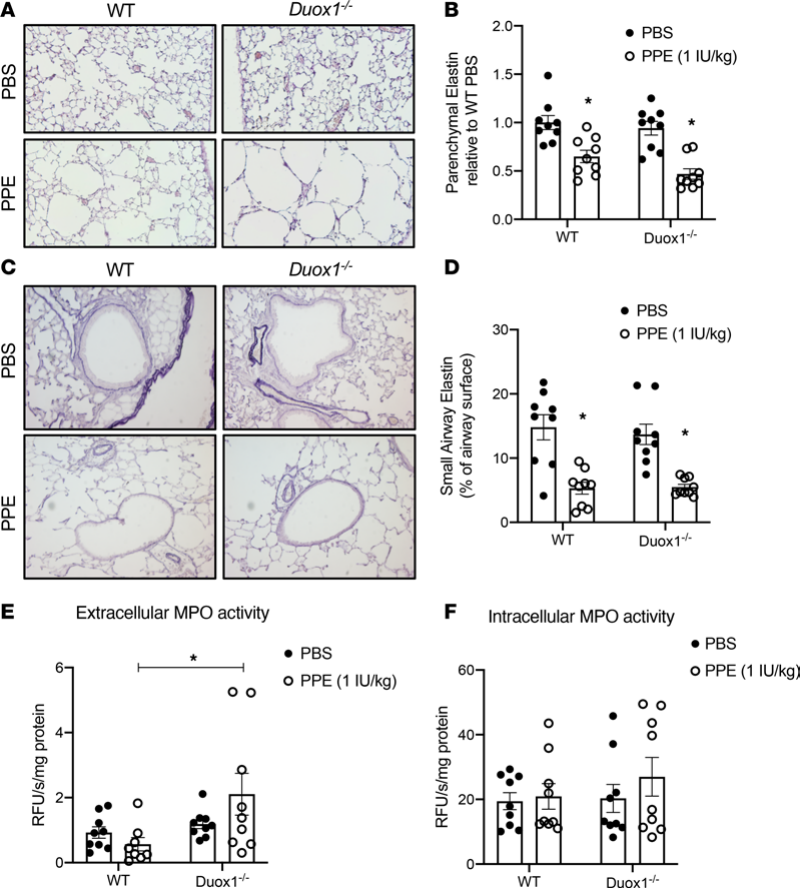
Development of elastase-induced emphysema is associated with decreased alveolar and small airway elastin levels, and *Duox1* deficiency enhances elastase-induced extracellular MPO activity. Analysis of parenchymal (**A** and **B**) and small airway (**C** and **D**) elastin levels (original magnification, ×200) in both WT and *Duox1^–/–^* mice in response to porcine pancreatic elastase (PPE) or PBS control with Weigert’s Resorcin Fuchsin staining. Analysis of extracellular (**E**) and intracellular (**F**) MPO activity in lung tissue homogenates. Data are shown as mean ± SEM; *n* = 9 per group, from 2 separate experiments. *P < 0.05 by 2-way ANOVA. MPO, myeloperoxidase.

To further explore a potential role for DUOX1 suppression in the development of COPD, we examined emphysema development in response to airway instillation of porcine pancreatic elastase (PPE), in age-matched WT and *Duox1^–/–^* C57BL/6NJ mice. As expected (41, 42), PPE exposure induced development of alveolar emphysema, as measured by increased alveolar airspace enlargement (Figure 5, A and B). Importantly, elastase-induced airspace enlargement in this model was significantly worsened in *Duox1^–/–^* mice (Figure 5, A and B), suggesting that the absence of DUOX1 increases susceptibility to elastase-induced emphysema. We observed reduced elastin levels in the remaining parenchymal tissue (Figure 6, A and B) and small airways (Figure 6, C and D) in response to elastase instillation in both WT and *Duox1^–/–^* mice. PPE-induced loss of parenchymal elastin appeared to be worsened in *Duox1^–/–^* mice (Figure 6B), but this was not statistically significant, and elastin degradation within the small airways was similar in WT and *Duox1^–/–^* mice (Figure 6D). Analysis of Picrosirius red staining indicated tendencies toward increased in small airway collagen in response to elastase, but this was not statistically significant in either WT (*P* = 0.1064) or *Duox1^–/–^* (*P* = 0.6932) mice (Supplemental Figure 4A). No significant increases were observed in parenchymal collagen levels in response to PPE (Supplemental Figure 4B). To gain further mechanistic insight, we evaluated lung tissue mRNA expression of extracellular matrix proteins (e.g., Collagen 1a1, elastin) and markers of inflammation and remodeling. Although several of these markers (*Col1a1*, *Eln*, *Mmp12*, *Il13*, *Cxcl1*) were significantly increased in elastase-exposed mice, no significant differences were observed between WT and *Duox1^–/–^* mice (Supplemental Figure 5). Since neutrophil infiltration plays an important role in emphysema development (43), we examined neutrophil activation by measuring intracellular and extracellular activity of the neutrophil granule protein myeloperoxidase (MPO) (44). Extracellular MPO activity levels in lung tissues from elastase-exposed *Duox1^–/–^* mice were significantly increased compared with corresponding WT mice, whereas intracellular MPO activity levels were unaffected (Figure 6, E and F), suggesting that *Duox1* deficiency promotes neutrophil activation and degranulation in this model of elastase-induced emphysema. Finally, based on previous findings implicating DUOX1 in airway production of amphiregulin (Areg), an important growth factor that contributes to epithelial regeneration after injury (45), we hypothesized that *Duox1* deficiency may lead to impaired Areg production in this model. Lung tissue Areg protein levels were similarly elevated in response to PPE in both WT and *Duox1^–/–^* mice (Supplemental Figure 6), even though lung tissue *Areg* mRNA tended to be suppressed in *Duox1^–/–^* mice treated with PPE compared with PBS controls (*P* = 0.0563).

## Discussion

Oxidative stress is often implicated in the pathogenesis of COPD, but findings on the involvement of NADPH oxidases (NOX) in experimental models of COPD are variable and sometimes even contradicting (17, 19–21). Our present findings extend intriguing previous observations that the primary epithelial NOX isoform DUOX1 is in fact downregulated within the airways of subjects with COPD (22, 23), and demonstrate a gradual loss of small airway DUOX1 protein expression in patients with COPD in correlation with lung function decline and extracellular matrix remodeling and emphysema. One limitation of our studies is that we were not able to perform stereology according to published guidelines (46), since we did not have serial sections available. Although DUOX1 was significantly downregulated in GOLD IV patients, it was not significantly reduced in patients with moderate COPD (GOLD II), which may suggest that DUOX1 status declines gradually as COPD progresses and may be a symptom of COPD pathology rather than a causative factor. In line with this argument, we observed that TGF-β, a feature of COPD pathology, can suppress DUOX1 within the airway. Alternatively, since DUOX1 downregulation may also be a result from smoking (22, 23), it is also plausible that DUOX1 downregulation due to smoking may contribute to COPD progression, or that lowered airway DUOX1 status at the onset of COPD development may actually enhance its progression. In support of this latter suggestion, we provide evidence that *Duox1* deficiency can worsen disease outcomes in 2 distinct mouse models that reflect different pathological hallmarks of COPD, i.e., small airway subepithelial fibrosis and alveolar airspace enlargement. These observations would therefore suggest that the gradual loss of DUOX1 in human COPD, potentially as a result of smoking (22), may be a contributing factor in COPD development and its progression. Unfortunately, our present studies were based only on current or former smokers and were not sufficiently powered to reveal a significant impact of smoking status on DUOX1. Previous studies have documented suppression of airway DUOX1 in active smokers compared with never smokers (22), consistent with our present findings using CSE or acrolein, but it is unclear whether this also persists in former smokers. Also, other studies have suggested that CSE exposure may actually enhance DUOX1 (47), and hence the precise relationship between smoking status and history and airway DUOX1 is complex. However, the fact that correlations between DUOX1 staining scores and lung function parameters were largely independent of smoking status (Figure 1B) would suggest that DUOX1 downregulation is associated with COPD severity and not with smoking history.

Outside the thyroid, DUOX1 is primarily expressed at mucosal surfaces, including the airway, and is thought to participate in oxidative mucosal host defense, analogous to the antimicrobial function of phagocyte oxidase. More recent studies demonstrated that DUOX1 contributes to innate epithelial and epidermal wound responses through redox-dependent activation of various cellular signaling pathways, and thereby contributes to maintenance of epithelial integrity (24). Downregulation of DUOX1 in COPD would therefore be expected to impair such innate lung injury responses and thereby result in impaired epithelial regenerative capacity. Of note, although our analysis of DUOX1 was largely based on analysis of small airways, DUOX1 is also present in the alveolar type II cells (48), where it likely plays similar roles in alveolar innate host defense and epithelial injury responses. Although our tissue stainings did not allow us to accurately quantify DUOX1 protein expression in the alveolar epithelium, we suspect that our observation of reduced small airway DUOX1 expression in COPD may also extend to similar DUOX1 downregulation in the alveolar epithelium of these patients. As a result, innate alveolar host defense and/or regenerative capacity may be diminished and lead to emphysema development in COPD.

Our recent studies have suggested that DUOX1 silencing, as is observed in many lung cancers, can lead to epithelial reprogramming with features of EMT (26), which may also be relevant for small airway remodeling in COPD (27). Indeed, chronic exposure to CS, which may be the primary cause of COPD, is well-known to promote EMT features (27, 49) and subsequent extracellular matrix remodeling and related thickening of the small airways (38, 40), and may also potentially result in impaired alveolar re-epithelialization (50). In our present studies we show that acrolein, a major component of CS, can similarly induce EMT features and small airway remodeling in mice, and that this was associated with *Duox1* downregulation and, more importantly, enhanced by *Duox1* deficiency. Thus, DUOX1 suppression during COPD may contribute to disease pathogenesis by enhancing EMT features and related airway remodeling.

To gain additional mechanistic insight into the impact of *Duox1* deficiency on acrolein-induced small airway remodeling as well as elastase-mediated airspace enlargement, we surveyed potential alterations in various markers of inflammation or remodeling that have previously been linked to DUOX1 in the context of innate airway injury responses or wound healing. These efforts unfortunately did not yield conclusive mechanistic insights, but in some cases showed surprising outcomes. For example, the matrix metalloproteinase Mmp-9, which was previously implicated in DUOX1-mediated epithelial wound responses (51) and has also been implicated in COPD as part of the protease/antiprotease imbalance (52), was found to be upregulated in response to chronic acrolein exposure, but this was similar in both WT and *Duox1*-deficient mice. The EGFR ligand Areg is produced as a critical mediator of epithelial regeneration during injury (45) through a pathway that may involve DUOX1 (53), although its importance in COPD is not well established. Our findings of PPE-induced emphysema suggested that lung tissue *Areg* mRNA tended to be suppressed in PPE-exposed *Duox1^–/–^* mice compared controls, but PPE-induced increases in lung tissue Areg protein levels were similar both WT and *Duox1^–/–^* mice (Supplemental Figure 6). It is unclear how such increase in Areg is relevant for emphysema, but upregulation of Areg in the airway basal cells in smokers has also been associated with basal cell and mucus hyperplasia (54), important features of COPD. Indeed, we observed increases in *Muc5ac* mRNA in PPE-treated mice, as well as *Il13*, an important mediator of mucus metaplasia and remodeling. PPE-induced upregulation of *Muc5ac* appeared to be further increased in *Duox1*-deficient mice (Supplemental Figure 5), which was unexpected in light of our previous observation that DUOX1 contributes to *Muc5ac* expression and mucus metaplasia in the context of allergic airway inflammation (53). These latter studies also indicated a critical role for DUOX1 in production of IL-13 during allergic inflammation, but *Il13* induction in the context of PPE-induced emphysema was unaltered in *Duox1*-deficient mice. These various differences in the apparent relationships between DUOX1, MMP-9, IL-13, Areg, or MUC5AC in these different contexts may be related to their different cellular source(s) for these mediators in these different disease models, whereas DUOX1 is likely involved only in epithelia-specific responses. Cell-specific analyses by, e.g., single-cell RNAseq would be required to more clearly dissect this. Intriguing recent studies demonstrated that IL-13 induction within the alveolar epithelium impairs self-renewal and differentiation properties of alveolar type 2 cells, which is likely relevant to alveolar remodeling and emphysema development (55).

Neutrophilic inflammation has been implicated in COPD pathology (56, 57) and in elastase-induced emphysema (41, 42), and previous studies of allergic airways disease have linked DUOX1 to production of neutrophil chemokines (CXCL1) and neutrophil recruitment (53, 58). However, we did not observe significant changes between WT and *Duox1^–/–^* mice with respect to overall neutrophil content during PPE-induced emphysema, based on intracellular MPO analysis, or with respect to induction of *Cxcl1* mRNA. However, analysis of extracellular MPO activity, which likely reveals neutrophil activation and degranulation, showed an increase particularly in PPE-exposed *Duox1*-deficient mice. The relationship between DUOX1 and neutrophilia is undoubtedly complex and also context dependent. For example, in contrast to observations during allergic airway inflammation, DUOX1 was not found to affect CXCL1 production and neutrophil recruitment in response to, e.g., LPS (59). Increased neutrophil degranulation in the context of *Duox1* deficiency may enhance tissue destruction, due to secretion of neutrophil-derived proteases and/or MPO-catalyzed oxidative activation of MMPs (60) or inactivation of tissue inhibitors of MMPs (61).

In summary, the current study highlights the potential importance of downregulation of airway (or alveolar) DUOX1 in the context of COPD, and indicates that it may be a contributing factor to COPD pathogenesis and progression. Although many questions remain with respect to the mechanisms involved, our observations suggest that DUOX1 downregulation can promote both small airway remodeling and alveolar airspace enlargement, both of which could be related to altered epithelial biology and homeostasis. We did not address the mechanism(s) by which DUOX1 is downregulated during COPD, but suggest that one factor could be activation of TGF-β, a signaling pathway that is commonly activated by CS exposure and has been strongly linked to COPD (62, 63). Alternatively, it is possible that epigenetic mechanisms, as seen in, e.g., lung cancer, may also contribute to DUOX1 silencing in COPD (64). Lastly, our findings have important implications for the popular notion of antioxidant-based approaches as a potential treatment of COPD, as these could also impair beneficial DUOX1-mediated redox mechanisms that promote innate airway defense or epithelial homeostasis. Instead, targeted approaches to prevent DUOX1 downregulation or enhance its function in the context COPD might in fact be more beneficial in managing this devastating disease and would deserve further exploration.

## Methods

### Human study subjects and tissue collection.

Lung tissues were obtained from the upper lobe subpleural area of 14 control, 16 GOLD II, and 19 GOLD IV patients at University Hospital Maastricht, as previously described by Eurlings et al. (28). Briefly, lung tissue sections of approximately 2 cm^2^ were obtained from GOLD IV patients undergoing LVRS, and tissues from control and GOLD II patients were obtained as tumor-free tissues during resection of a solitary primary tumor. Other details regarding tissue collection, exclusion criteria, smoking history, and lung function analysis were performed as previously described (28), and the same paraffin-embedded tissue sections were also used in the present study.

### SPC-TNF-α model.

SPC-TNF-α mice (*n* = 10, male and female), which are transgenic mice that exhibit chronic pulmonary inflammation resulting from overexpression of TNF-α in alveolar epithelial type II cells (TNF-α expression under the control of the promoter of SP-C; expressed by alveolar epithelium type II), were euthanized at 6 months of age, and lungs were harvested and paraffin embedded as previously described (32). Various readouts were compared between SPC-TNF-α mice and age-matched male and female transgene negative littermates (WT, *n* = 10).

### Acrolein model.

WT male and female C57BL6/J mice aged 8–12 weeks and age-matched male and female *Duox1^–/–^* mice, originally generated on C5757BL6/J background and provided by Miklos Geiszt (65), were subjected to chronic acrolein exposure, as described previously (66). Briefly, mice were placed in a 2 L glass chamber and exposed to either 5 ppm (11.5 mg/m^3^) of acrolein vapor or control air, for 4 hours/day, 5 days/week, for 2 weeks total. Mice were euthanized after the final exposure, and lung tissues were collected for analysis of various readouts.

### Elastase (PPE) model.

WT male and female C57BL6/NJ mice aged 8–12 weeks and corresponding age-matched male and female *Duox1^–/–^* mice (backcrossed to C57BL6/NJ mice; Jackson Laboratories) were subjected to oropharyngeal instillation of porcine pancreatic elastase (PPE; 1 IU/kg bodyweight in 50 μl PBS; Elastin Products Company, EC134) or 50 μl PBS vehicle control, under brief isoflurane anaesthesia, which was repeated once a week for a total of 3 weeks. One week after the final instillation, mice were euthanized, and lung tissues were collected for analysis of mean linear intercept (MLI), as well as other outcomes.

### Lung tissue fixation and IHC.

Mouse lung tissues were collected upon completion of the indicated experiments, and left lung lobes were fixed in paraformaldehyde (PFA) and paraffin embedded for IHC. For MLI analysis, the lungs in the PPE model were fixed by tracheal instillation of 4% PFA at a pressure of 25 cm H_2_O for 20 minutes. Only sections that displayed no cutting artifacts, compression, or hilar structures were used in the MLI analyses.

Tissue sections (5 μm) were cut and stained with either H&E or Masson’s trichrome (MTA) using standardized protocols after deparaffinization. Additionally, fixed sections were immunohistochemically stained for α-smooth muscle actin (1:8000; MilliporeSigma, A2547), detected using Vectastain Alkaline Phosphatase Universal, Vector Red (Vector Laboratories). For elastin staining, slides were incubated for 20 minutes in Weigert’s Resorcin-Fuchsin (Electron Microscopy Sciences) at 60°C to 70°C. Collagen was stained by incubation for 90 minutes in 0.1% Sirius Red in saturated picric acid (Electron Microscopy Sciences).

Paraffin-embedded tissue sections from non-COPD control subjects and GOLD II and GOLD IV patients (4 μm thickness) were evaluated for the presence of the DUOX1 protein using a DUOX1 antibody (1:500; Santa Cruz Biotechnology, SC48858) and visualized utilizing a biotin-conjugated secondary antibody (Dako, E0466), the Vectastain Peroxidase ABC Kit, and Enzyme Substrate (Vector Blue; Vector Laboratories), with Nuclear Fast Red counterstaining. Small-to-medium sized airways, defined as smaller than 2 mm in diameter, were scored for staining of DUOX1 based on a scoring scale of 1–4 (Supplemental Figure 1), in which 4 was the highest staining intensity, and a score of 1 was the lowest staining intensity (minimal staining observed). Negative staining controls were performed by omission of the primary antibody. Two independent researchers, blinded to the tissue identity, quantified the DUOX1 scoring in 2–4 airways per tissue section to obtain an average small airway DUOX1 staining score for each section, after which the individual staining scores for each observer were averaged, thus obtaining mean scores that are asymptotically continuous and normally distributed. Lung tissue sections of the SPC-TNF-α mice were evaluated similarly for the presence of small airway Duox1 protein as described above (antibody dilution 1:200, SC48858).

Stainings for elastin, collagen, and α-SMA were quantified using MetaMorph imaging software (Molecular Devices). MTA stainings were quantitatively scored as described previously (53).

### Quantification of airspace enlargement.

Enlargement of alveolar spaces was determined by quantifying the MLI using Stereo-Investigator software (MBF Bioscience). For each lung, 4–5 images were analyzed, with a minimum of 50 measurements per image. Briefly, MLI was measured by first placing 40 μm spacing between lines over the tissue sections and consequently marking points (P) on the alveoli airspace to estimate volume, and intersections (I) were marked on the alveolar walls to estimate surface. The MLI was then calculated according to previously established methods (67), using the formula MLI = 2 × *k* × *d* × *P*/*I*, in which *k* is the length of line used to probe, *d* is number of lines per point, *P* is the number of points marked in the alveoli airspaces, and *I* is number of intersections marked between the probe lines and the surface of the alveoli.

### MPO assay.

MPO activity in lung tissues was measured according to the step-by-step protocol (44). Briefly, lungs were placed in extraction buffer (0.32 M sucrose, 1 mM CaCl_2_, 10U/ml heparin in HBSS) for 2 hours on ice to extract extracellular proteins. After incubation, the supernatant was transferred, precipitated, and resuspended in PBS (extracellular fraction). The lungs were then placed in CTAB buffer (50 mM cetyltrimethylammonium bromide in 50 mM potassium phosphate buffer at pH  =  6) and were subsequently homogenized, sonicated, and freeze-thawed in liquid nitrogen. After centrifugation, supernatant was collected, representing the intracellular protein fraction. After extraction of both intracellular and extracellular proteins, MPO was captured using MPO ELISA dilution buffer (Hycult) on anti-MPO antibody-coated plates (Hycult) for 1 hour at room temperature. Assay wells were then washed, and MPO activity of antibody-captured MPO was assessed with 10-acetyl-3,7-dihydrophenoxazine according to protocol (44).

### Cell culture.

PBECs, kindly provided by the Primary Lung Culture facility of the Maastricht University (MU) Medical Center, were isolated from lung tissues resected during lobectomies or pneumonectomies of patients who underwent surgery for lung cancer. PBECs of 3 donors without known history of chronic lung disease were isolated and cultured as previously described (68). Upon confluence, cells were starved overnight, after which cells were exposed to varying concentrations of CSE (1%, 2%, or 4%) for 24 hours. 3R4F Research Cigarettes (University of Kentucky, Lexington, Kentucky, USA) were removed from their filters and CSE was prepared in HBSS as previously described (69).

In addition, primary MTECs were isolated from excised mouse tracheas from either WT mice or *Duox1^–/–^* mice (C57Bl6/J) and cultured as previously described (25) and used for in vitro experiments.

### ELISA.

Cell culture supernatants or BAL fluids were analyzed for TGF-β and Areg using DuoSet ELISA′s (R&D Systems) according to the manufacturer’s instructions.

### Western blot analysis.

Cell lysates were prepared using Western solubilization buffer (50 mM HEPES, 250 mM NaCl, 1.5 mM MgCl_2_, 1% Triton X-100, 10% glycerol, 1 mM EGTA, 1 mM PMSF, 2 mM Na_3_VO_4_, 10 μg/ml aprotinin, 10 μg/ml leupeptin; pH 7.4). Samples containing equal amounts of protein (BCA Protein Assay Kit; Pierce) were separated on 10% SDS-PAGE gels, transferred to nitrocellulose membranes, and probed with antibodies against Vimentin (1:500; Cell Signaling, 5741), β-actin (1:5.000; MilliporeSigma, A5316), or E-cadherin (1:1000; Cell Signaling, 3195). Antibodies were probed with rabbit- or mouse-specific secondary antibodies (Cell Signaling) conjugated with HRP and detected by chemiluminescence using SuperSignal West Pico Chemiluminescent Substrate (Pierce).

### qPCR analysis.

Target gene expression in lung tissues was analyzed by qPCR and normalized to GAPDH using the ddCT method. RNA was purified according to the GeneJET RNA Purification Kit (Thermo Fisher Scientific); first-strand cDNA was synthesized from purified RNA (1 μg) using an M-MLV Reverse Transcriptase Kit (Invitrogen). Real-time PCR (qPCR) reactions contained cDNA (0.5 μL), iQ SYBR Green Supermix (5 μL; Bio-Rad), and primer (1 μL) (Supplemental Table 4) in ddH_2_O (100 nM final) and ddH_2_O (3.5 μL). Amplification and detection were performed using a CFX96 Real-Time PCR Detection System (Bio-Rad). The following qPCR procedure was used: Preincubation for 3 minutes at 95°C, followed by 40 cycles of denaturation at 95°C for 5 seconds, annealing at 60°C for 1 minute, and amplification at 72°C for 30 seconds. A post-PCR melt curve was performed at 95°C for 10 seconds, followed by a 0.5°C incremental increase every 5 seconds from 65°C to 95°C. For the SPC-TNF mouse model and human PBECs, RNA was purified using the High Pure RNA Isolation Kit (Roche); first-strand cDNA was synthesized from purified RNA (1 μg) using the Transcriptor cDNA Synthesis Kit (Roche). qPCR reactions contained SensiMix SYBR Hi-ROX Kit (Quantace-Bioline) with primers (300 nM) and were performed in a 384-well MicroAmp Optical 384-Well Reaction Plate (Applied Biosystems) on a 7900HT Fast Real-Time PCR System (Applied Biosystems). The expression of the genes of interest was normalized with a correction factor derived by GeNorm (70), based on the expression RPL13A as reference gene.

### Statistics.

All quantitative data, unless specifically indicated, are presented as the mean ± SEM. Statistical differences between groups were analyzed using 2-way ANOVA with Tukey’s post hoc analysis in GraphPad Prism (version 8.3.0). Patient characteristics data from the human COPD cohort are displayed as mean ± SD and were normally distributed. Basic characteristics were analyzed using ANOVA or χ^2^. DUOX1 staining in this cohort was analyzed using ANCOVA using age, sex, pack-years, and smoking status as covariates with Bonferroni’s post hoc analysis. The number of current (12) and former (30) smokers provided 80% power to detect differences in DUOX1 due to smoking status of 0.7 or larger. In DUOX1 staining scores, among current smokers, the study had 80% power to detect differences greater than or equal to 1.7 between control and GOLD II patients; whereas among former smokers, there was 80% power to detect differences greater than or equal to 1.0 between control and GOLD II patients and differences greater than or equal to 0.7 between control and GOLD IV patients. Correlations between DUOX1 staining scores and other parameters were analyzed by Pearson’s correlations, with 2-tailed significance. A *P* value of less than 0.05 was considered significant.

### Study approval.

All animal procedures conducted at University of Vermont were reviewed and approved by the Animal Care and Use Committee at University of Vermont. Animal procedures conducted at MU were approved by the Institutional Animal Care Committee at UM. Collection, storage, and use of tissue and patient data were performed in agreement with the “Code for Proper Secondary Use of Human Tissue in the Netherlands”. The scientific board of the Maastricht Pathology Tissue Collection (MPTC) approved the use of materials for this study (MPTC2010-019). Formal permission was obtained from the local medical ethic committee (2017-0087) at MU, and patients provided written informed consent to permit the use of the material for research.

## Author contributions

CS and CVDW performed the analysis of human tissues and primary human cell cultures. CS, RAB, AH, and MH performed and analyzed the animal studies. SL, CMD, and MH contributed to data analysis. NLR and EFMW assisted with experimental design and analysis of human COPD studies. PMV assisted with statistical analyses. AVDV was responsible for the conception and overall supervision of the project and the final version of the manuscript. CS wrote the draft of the manuscript. All authors contributed to the planning, discussion, and interpretation of experiments and the writing of the manuscript.

## Supplementary Material

Supplemental data

## Figures and Tables

**Table 1:**
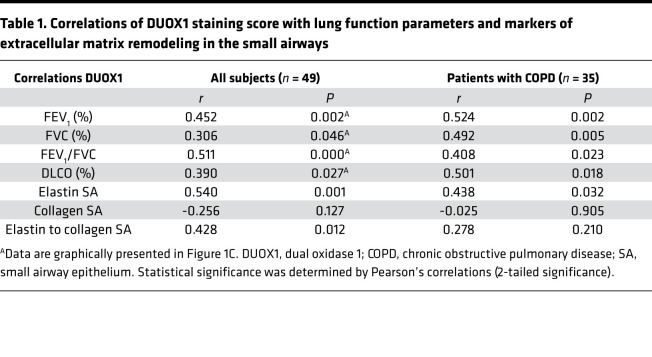
Correlations of DUOX1 staining score with lung function parameters and markers of extracellular matrix remodeling in the small airways

## Version 1 12/10/2020

In-Press Preview

## Version 2 01/25/2021

Electronic publication
