# Supplementary material for: Downregulation of epithelial DUOX1 in chronic obstructive pulmonary disease
Source: JCI Insight. 2021 Jan 25;6(2):e142189. doi: 10.1172/jci.insight.142189 (PMC7934842; doi:10.1172/jci.insight.142189)

Supplemental Information for:

Downregulation of Epithelial DUOX1 in Chronic Obstructive Pulmonary Disease Contributes to Disease Pathogenesis

Caspar Schiffers, Cheryl van de Wetering, Robert A. Bauer, Aida Habibovic, Milena Hristova, Christopher M. Dustin, Sara Lambrichts, Pamela M. Vacek, Emiel F.M. Wouters, Niki L. Reynaert, Albert van der Vliet*

Supplemental Table 1:

Clinical characteristics of study subjects

	Control	GOLD II	GOLD IV
**Number**	14	16	19
**Age (yrs)**	63 ± 6	65 ± 7	61 ± 7
**Sex (M/F)**	9/5	13 / 3 * $	12/7
**Pack-years [a]**	25 ± 6	46 ± 14 *	47 ± 29 *
**Smoking status (current/ex)**	4/9 [c]	11/5	0/19 * #
**FEV1 (% predicted)**	107 ± 15	67 ± 9 *	22 ± 4 * #
**FEV1/FVC**	80 ± 6	59 ± 9 *	26 ± 7 * #
**Dlco (% predicted) [b]**	95 ± 16	77 ± 18 *	35 ± 15 * #

*Data displayed as mean ± SD or ratio. * $p < 0.05$ compared to controls. # significant compared to GOLD II. $ significantly different compared to GOLD IV. [a]Values from 3 controls, 4 GOLD II and 2 GOLD IV patients missing. [b]Values from 2 controls, 1 GOLD II and 9 GOLD IV patients are missing. [c]Pack-years from 1 control missing. FEV1 and FEV1/FVC are measured post-bronchodilation. Patient characteristics were analysed using ANOVA or Chi Square.*

Supplemental Table 2: Detailed statistics of DUOX1 score

T-test for differences in DUOX1 scores for current and former smokers.						
**Smoking status**	**N**	**Mean**	**Std Dev**	**Std Err**	**Minimum**	**Maximum**
Current	12	2.25	0.7385	0.2132	1.25	3.5
Former	30	2.00	0.6979	0.1274	1	4

**T-test Results**		
**DF**	**t Value**	**P-value**
40	1.03	0.3083

ANOVA for differences in DUOX1 between controls, GOLD II and GOLD IV former smokers						
**Group**	**N Obs**	**Mean**	**Std Dev**	**Std Err**	**Minimum**	**Maximum**
Control	**8**	2.53	0.6187	0.2188	2	3.75
GOLD II	**4**	2.31	1.2479	0.6240	1.25	4
GOLD IV	**18**	1.96*	0.3792	0.0894	1	2.5

*P < .01 for GOLD IV vs. Control

T-test for differences in DUOX1 scores for controls and GOLD II for current smokers.						
**Smoking status**	**N**	**Mean**	**Std Dev**	**Std Err**	**Minimum**	**Maximum**
Control	3	2.33	1.0408	0.6009	1.5	3.5
GOLD II	9	2.22	0.6897	0.2299	1.25	3.5

**T-test Results**		
**DF**	**t Value**	**P-value**
10	0.22	0.8336

Supplemental Table 3: Bivariate correlations between DUOX1 and other variables

Bivariate correlations between DUOX1 and other variables

	Pearson Correlation Coefficients							Spearman Correlation Coefficients						
	**age**	**bmi**	**FVC**	**Dlco**	**FEV1**	**FEV1/FVC**	**Packyrs**	**age**	**bmi**	**FVC**	**Dlco**	**FEV1**	**FEV1/FVC**	**Packyrs**
**n**	42	42	43	32	43	43	35	42	42	43	32	43	43	35
**rho**	0.194	0.206	0.306	0.390	0.452	0.511	-0.083	0.204	0.295	0.445	0.476	0.467	0.479	-0.065
**p-value**	0.2191	0.1909	0.0456	0.0272	0.0024	0.0005	0.6353	0.1954	0.0575	0.0028	0.0059	0.0016	0.0012	0.7121

After log transform of DUOX1							
**n**	42	42	43	32	43	43	35
**rho**	0.205	0.236	0.362	0.421	0.466	0.496	-0.070
**p-value**	0.1918	0.1321	0.0172	0.0163	0.0016	0.0007	0.6906

Partial correlations with DUOX1 after adjustment for other variables

	**FVC**	**Dlco**	**FEV1**	**FEV1/FVC**	**FVC**	**Dlco**	**FEV1**	**FEV1/FVC**
Adjusted for smoking status								
**n**	42	31	42	42	42	31	42	42
**rho**	0.281	0.412	0.452	0.510	0.428	0.490	0.453	0.461
**p-value**	0.0749	0.0236	0.003	0.0007	0.0052	0.006	0.003	0.0024
Adjusted for pack years								
**n**	35	25	35	25	35	25	35	25
**rho**	0.326	0.425	0.491	0.546	0.484	0.496	0.474	0.451
**p-value**	0.06	0.0385	0.0032	0.0008	0.0037	0.0136	0.0046	0.0075
Adjusted for age								
**n**	42	32	42	42	42	32	42	42
**rho**	0.270	0.369	0.405	0.483	0.430	0.468	0.438	0.445
**p-value**	0.0881	0.0445	0.0085	0.0014	0.005	0.0091	0.0042	0.0035
Adjusted for BMI								
**n**	42	32	42	42	42	32	42	42
**rho**	0.333	0.295	0.468	0.521	0.476	0.375	0.482	0.473
**p-value**	0.0333	0.1068	0.002	0.0005	0.0016	0.0378	0.0014	0.0018

Supplemental Table 4: qPCR primers used in studies

Target gene	Forward/ Reverse primer	Primer sequence
DUOX1 (human)	F	TTCACGCAGCTCTGTGTCAA
	R	AGGGACAGATCATATCCTGGCT
RPL13A (human)	F	CCTGGAGGAGAAGAGGAAAGAGA
	R	TTGAGGACCTCTGTGTATTTGTCAA
Duox1	F	ACCAGAACATTGCGATGTATGAG
	R	AGAAATGGACGGTATCCTGGA
Gapdh	F	CTGGAGAAACCTGCCAAGTA
	R	TGTTGCTGTAGCCGTATTCA
Col1a1	F	CACCCTCAAGAGCCTGAGTC
	R	AGACGGCTGAGTAGGGAACA
Cxcl1	F	GTG AAT CAA GAC ATA GTT AAC C
	R	GTG AAT CAA GAC ATA GTT AAC C
Il6	F	AACGATGATGCACTTGCAGA
	R	GGAAATTGGGGTAGGAAGG
Areg	F	AAC GGT GTG GAG AAA AAT CC
	R	TTG TCC TCA GCT AGG CAA TG
Eln	F	TCCTGGAGCCACTCTTACAG
	R	CTCTCTCTCCCCAATTAGCC
Acta2	F	CGCTGTCAGGAACCCTGAGA
	R	CGAAGCCGGCCTTACAGA
Mmp9	F	CTCACTCACTGTGGTTGCTG
	R	TGGTTATCCTTCCTGGATCA
Mmp12	F	TTTCTTCCATATGGCCAAGC
	R	GGTCAAAGACAGCTGCATCA
Il1b	F	GCCCATCCTCTGTGACTCAT
	R	AGGCCACAGGTATTTTGTCG
Il13	F	CTACAGCTCCCTGGTTCTCT
	R	TTGCTCAGCTCCTCAATAAG
Tgfb	F	TGC TTT AGC TCC ACA GAG AA
	R	TGG TTG TAG AGG GCA AGG AC
Muc5ac	F	AGTCTCTCTCCGCTCCTCTCAAT
	R	CAGCCGAGAGGAGGGTTTGATCT

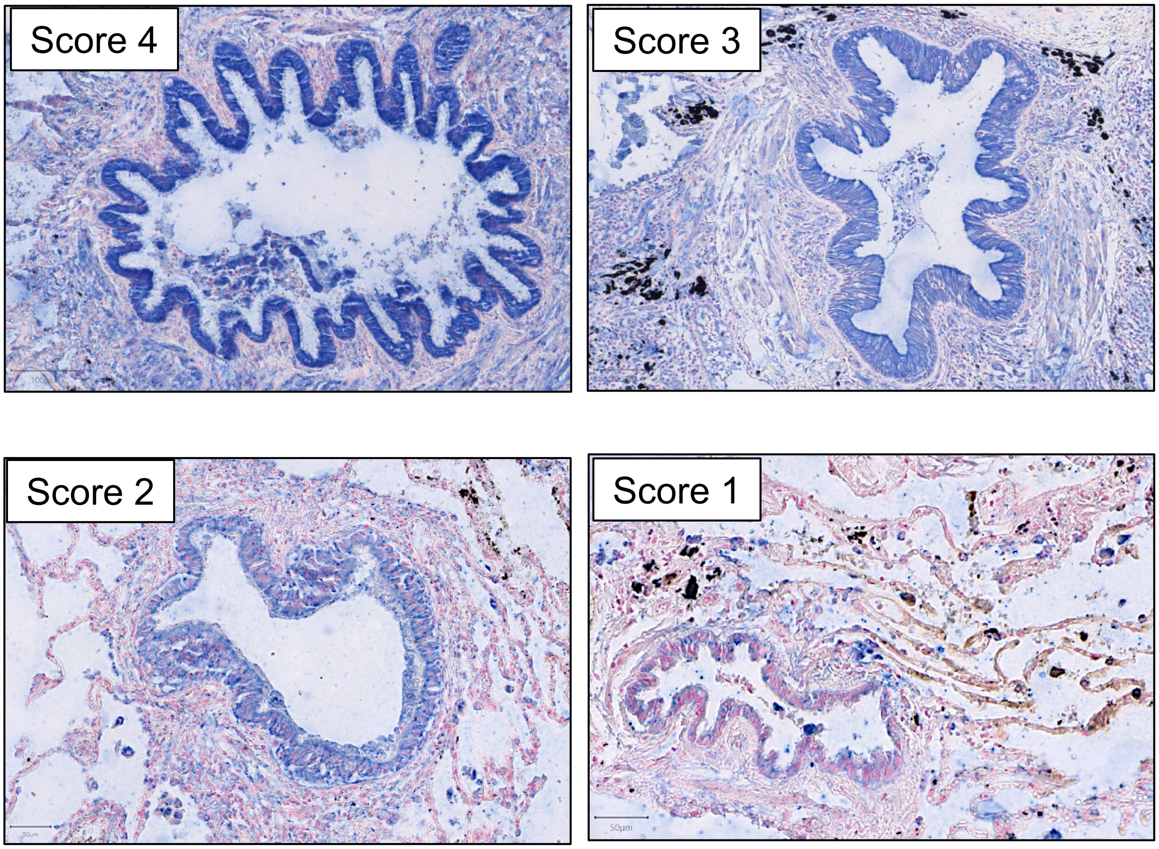

Supplemental Figure 1: Definition of the small Airway IHC scores 1, 2, 3 and 4 for DUOX1 (blue staining) for control, GOLDII and GOLDIV COPD patients (100x magnification).

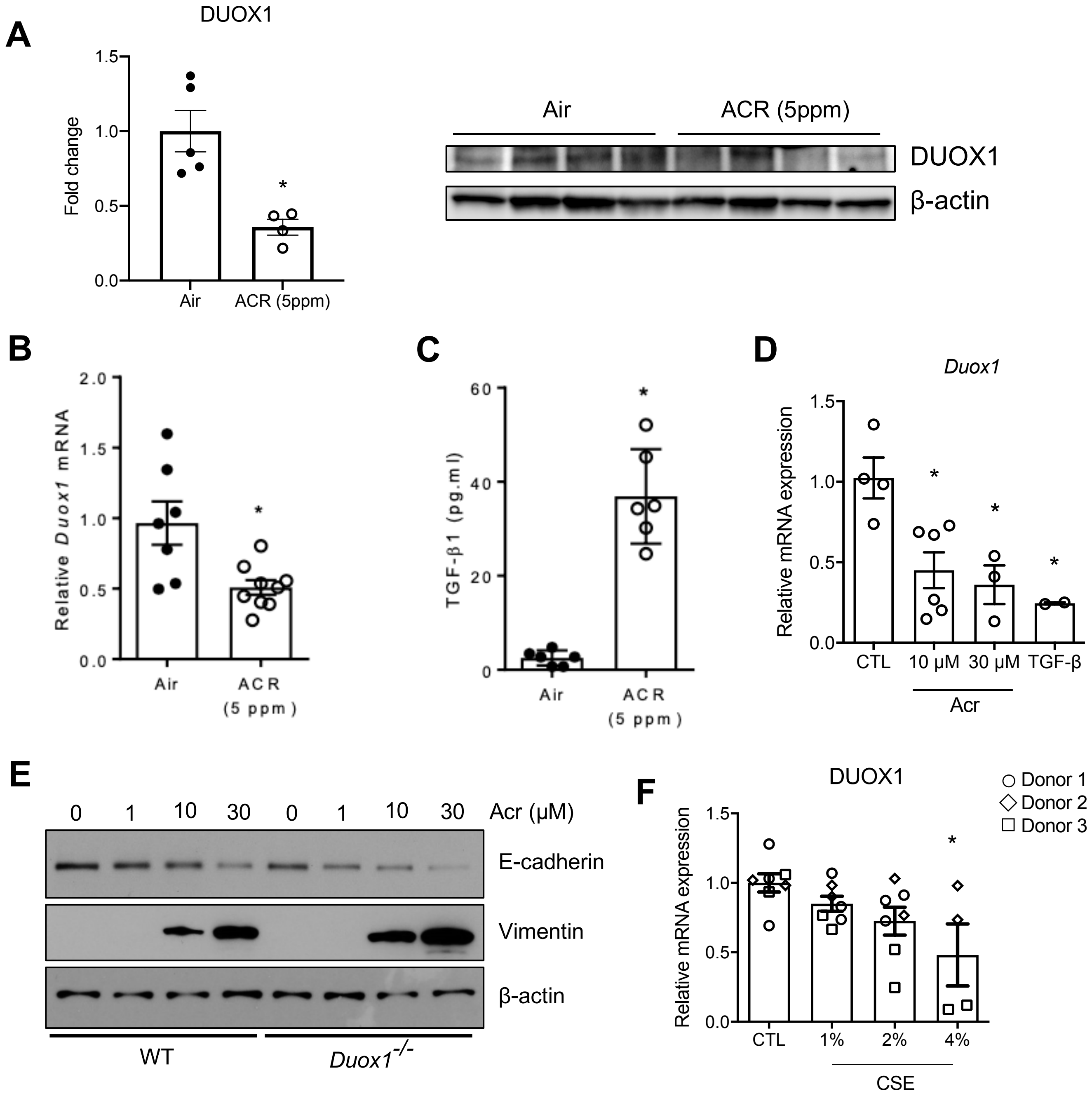

Supplemental Figure 2: DUOX1 (protein and/or mRNA) expression is reduced in a mouse model of chronic acrolein exposure, in isolated mouse tracheal epithelial cells (mTEC) exposed to acrolein or TGF-β, and in primary bronchial epithelial cells (PBEC) exposed to cigarette smoke. Lung tissue *Duox1* protein (A, n=4-5, with representative Western Blot) and mRNA (B, n=6-9) and C) BAL fluid TGF-β1 levels (n=6-9) in mice exposed to acrolein (5 ppm; 4 hrs/day, for 2 weeks) or air. C) Cultured mTECs from WT (and *Duox1*$^{-/-}$ mice in (D)) were exposed daily to acrolein (1 - 30 μM) or TGF-β (10 ng/ml) for 14 days and analyzed for *Duox1* mRNA or D) E-cadherin and vimentin by Western blot. E) DUOX1 mRNA expression in isolated PBECs from non-COPD patients 24 hours after treatment with 1,2 or 4% cigarette smoke extract (CSE); Values from 2-3 different donors, as indicated by symbols. Data shown as mean ± SE. n=6-10; *: $p < 0.05$, by 1-way ANOVA, or 2-tailed unpaired *t*-test.

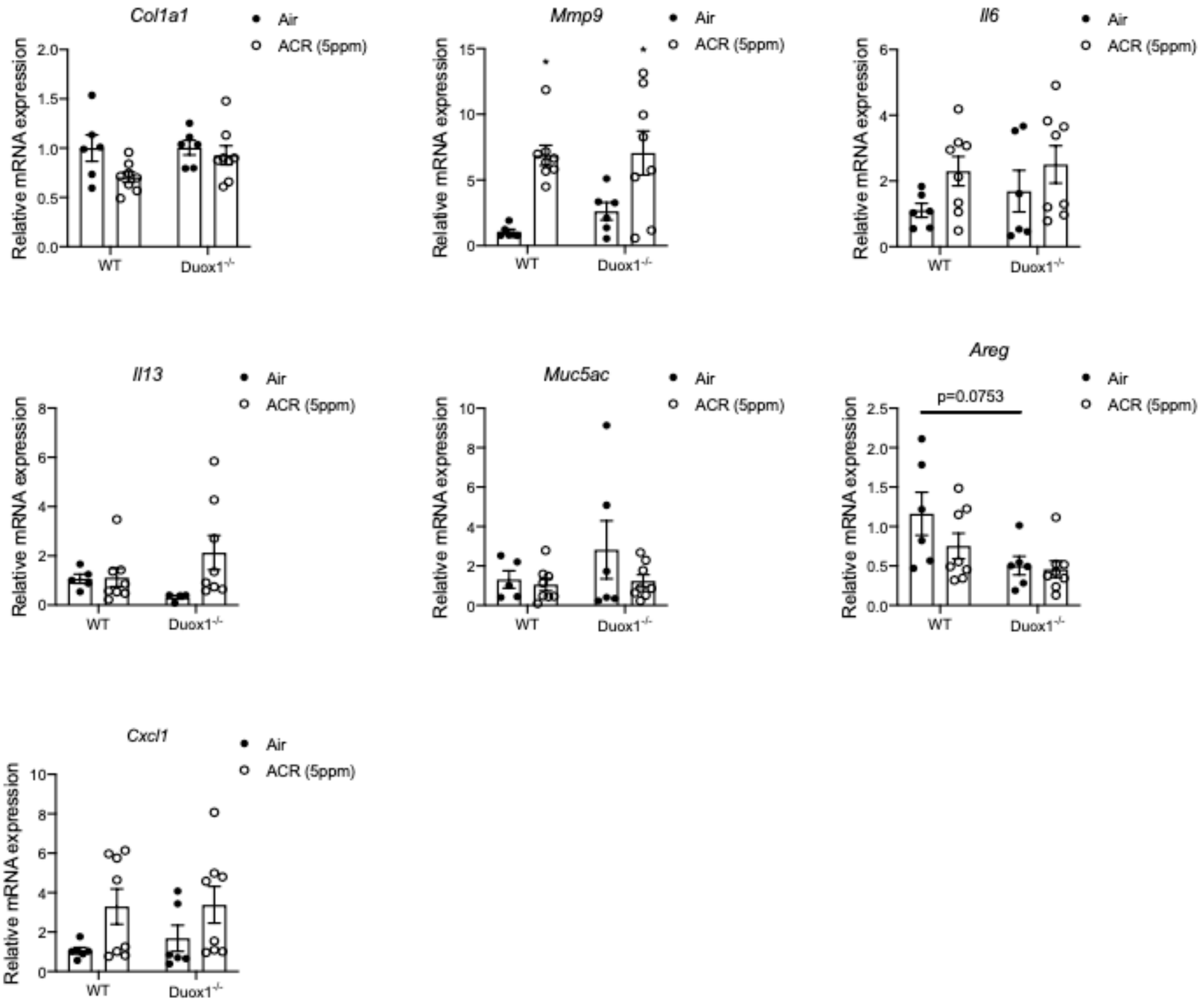

Supplemental Figure 3: mRNA analysis of various markers of matrix remodeling, tissue regeneration, and tissue inflammation in WT and *Duox1*$^{-/-}$ mice exposed to acrolein (5 ppm; 4 hrs/day, for 2 weeks) or air. Data shown as mean ± SE. n= 4-8. * p < 0.05, by 2-way ANOVA.

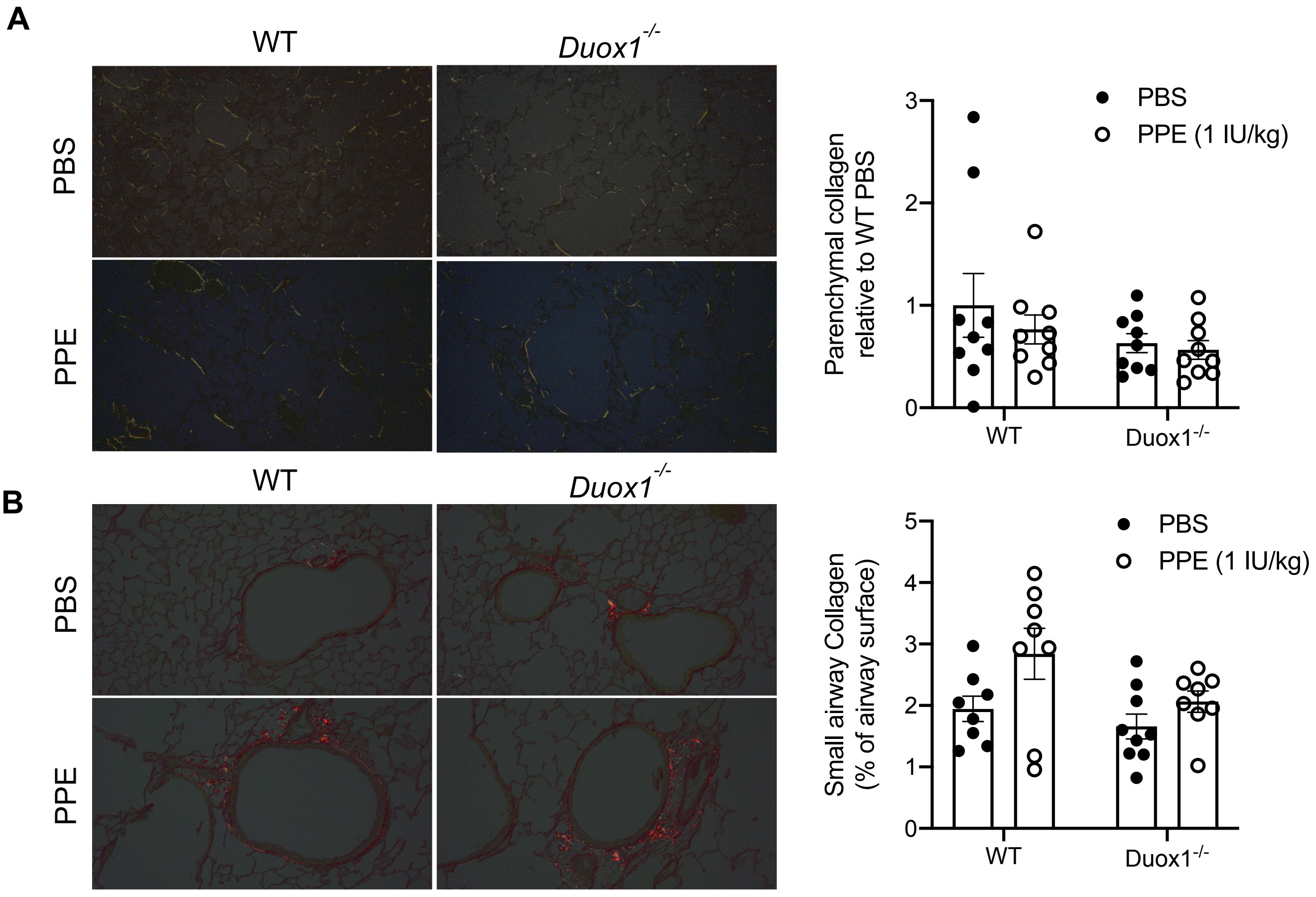

Supplemental Figure 4: Small airway and parenchymal collagen levels are unaltered in WT and *Duox1*$^{-/-}$ mice exposed to porcine pancreatic elastase (PPE). Small airway (A) and parenchymal (B) collagen levels were determined in both WT and *Duox1*$^{-/-}$ mice in response to porcine pancreatic elastase or PBS control with Picrosirius Red staining and quantification (200x magnification). Data shown as mean ± SE. n= 8-9.

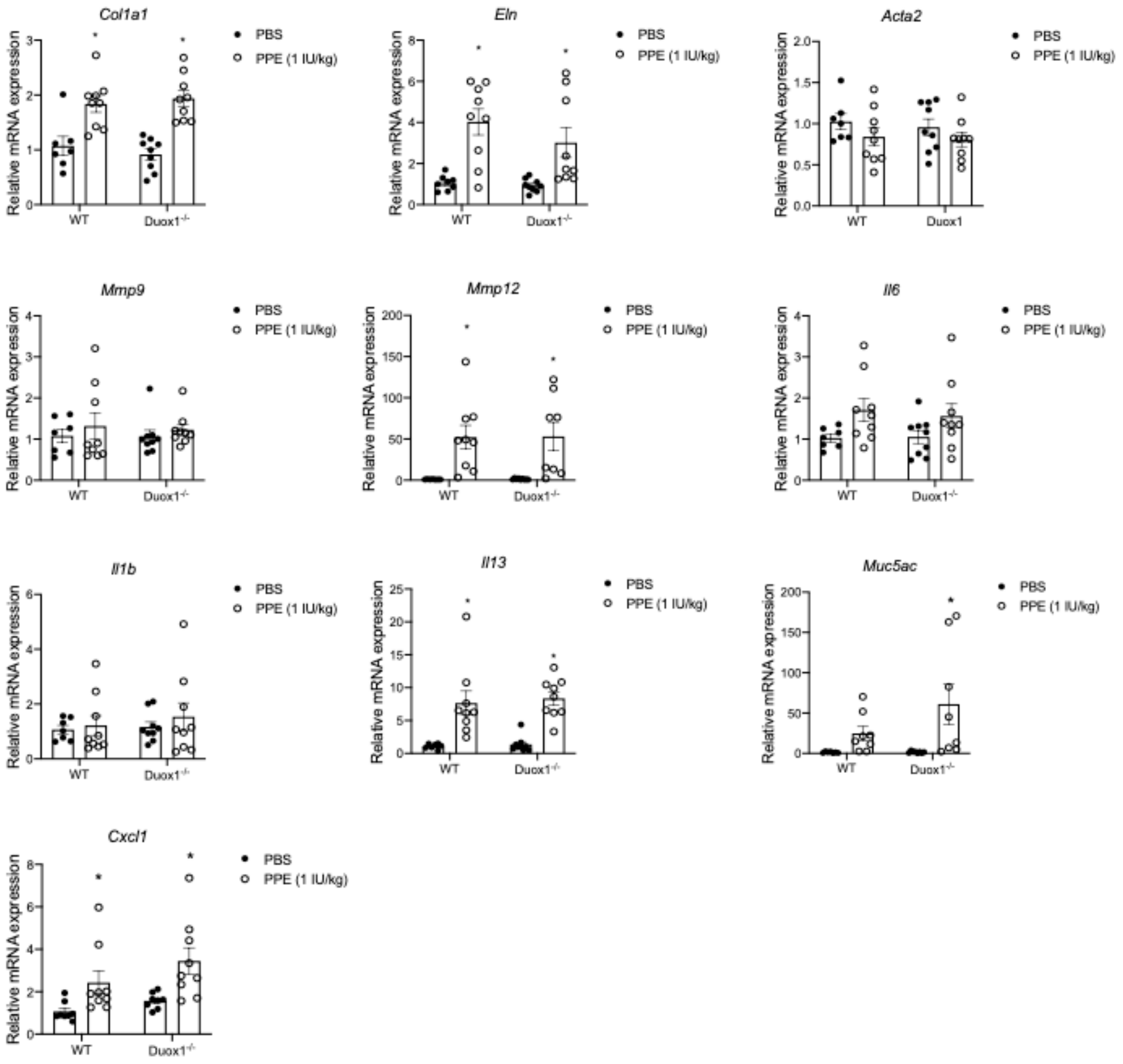

Supplemental Figure 5: mRNA analysis of various markers of matrix remodeling, tissue regeneration, and tissue inflammation in WT and *Duox1*$^{-/-}$ mice exposed to 50 μL porcine pancreatic elastase (PPE, 1 IU/kg bodyweight, 1 day/week, for 3 weeks) or PBS control. Data shown as mean ± SE. n=8-9; * $p < 0.05$, by 2-way ANOVA.

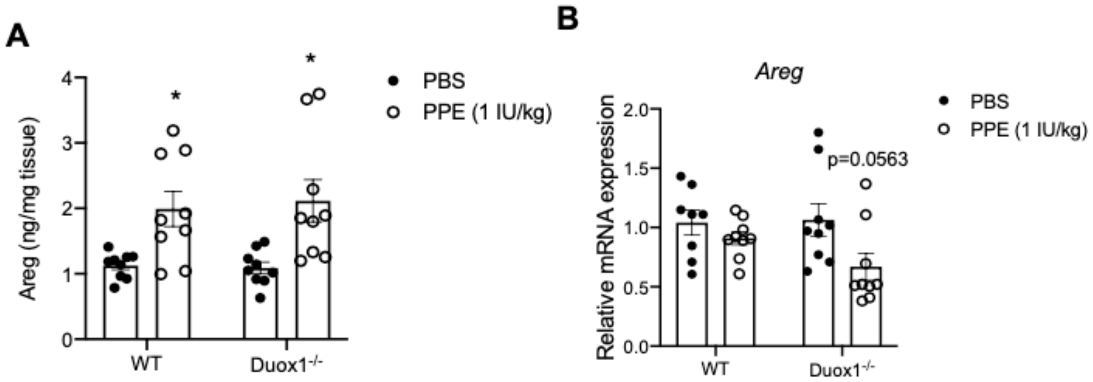

Supplemental Figure 6: Areg mRNA is suppressed in *Duox1*$^{-/-}$ deficient mice upon elastase exposure, but whole lung tissue levels are similarly upregulated in WT and *Duox1*$^{-/-}$ mice. A) lung tissue Areg protein (ng/mg) and B) mRNA expression in WT and *Duox1*$^{-/-}$ mice exposed to 50 μL porcine pancreatic elastase (PPE, 1 IU/kg bodyweight, 1 day/week, for 3 weeks) or PBS control. Data shown as mean ± SE. n=8-9; * $p < 0.05$, by 2-way ANOVA.

Full unedited gel for Figure Supplement 1D

E-cadherin: cell signaling #5741

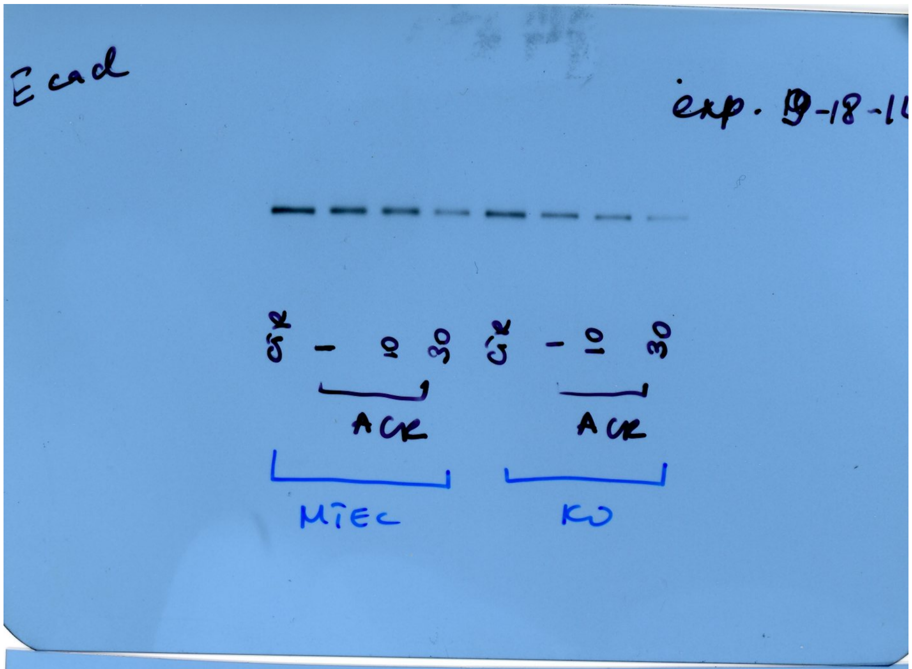

Vimentin: cell signaling #3195

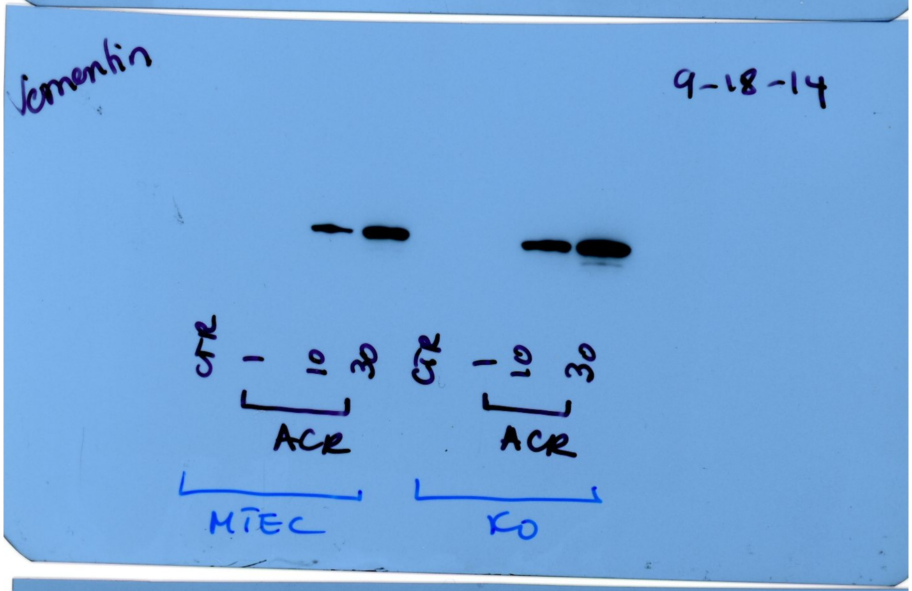

B-actin Sigma A5316

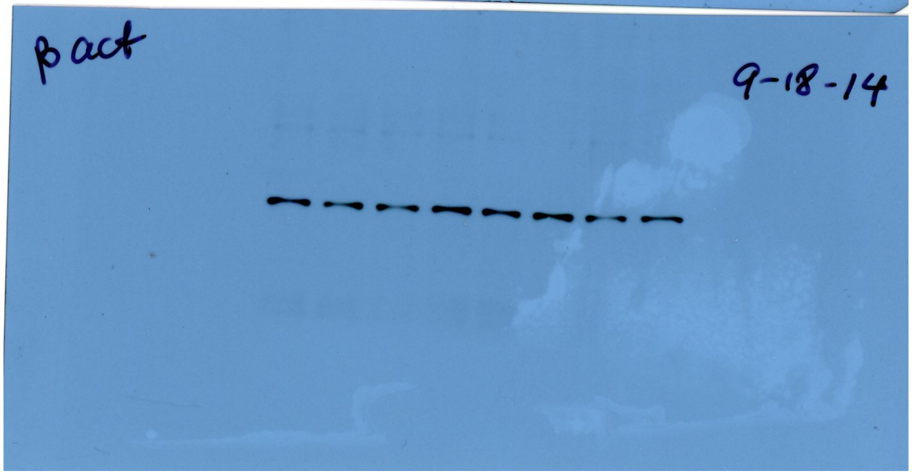

Full unedited gel for Figure S1A

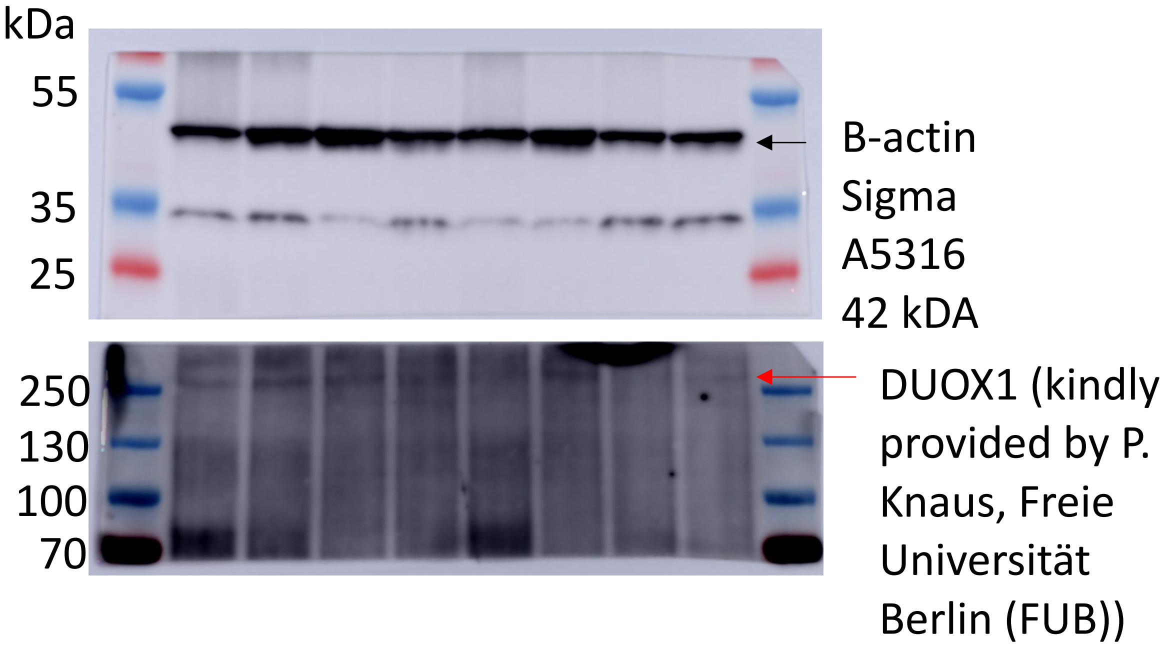

Supplement: Supplemental data [file jciinsight-6-142189-s008.pdf]
